# Molecular Interactions of the Polysialytransferase Domain (PSTD) in ST8Sia IV with CMP-Sialic Acid and Polysialic Acid Required for Polysialylation of the Neural Cell Adhesion Molecule Proteins: An NMR Study

**DOI:** 10.3390/ijms21051590

**Published:** 2020-02-26

**Authors:** Si-Ming Liao, Bo Lu, Xue-Hui Liu, Zhi-Long Lu, Shi-Jie Liang, Dong Chen, Frederic A. Troy, Ri-Bo Huang, Guo-Ping Zhou

**Affiliations:** 1 The National Engineering Research Center for Non-Food Biorefinery, Guangxi Academy of Sciences, Nanning 530007, Guangxi, China; simingliao@gxas.cn (S.-M.L.); lubo@gxas.cn (B.L.); luzhilong@gxas.cn (Z.-L.L.); zhongli57301@gmail.com (S.-J.L.); chendong@gxas.cn (D.C.); 2 Institute of Biophysics, Chinese Academy of Sciences, Beijing 100101, China; xhliu@moon.ibp.ac.cn; 3 Department of Biochemistry and Molecular Medicine, University of California School of Medicine, Davis, CA 95616-8635, USA; 4 College of Life Science and Technology, Guangxi University, Nanning 530004, Guangxi, China; 5 Gordon Life Science Institute, Rocky Mount, NC 27804, USA

**Keywords:** NMR, sialic acid (Sia), polysialic acid (polySia), polysialyltransferases, ST8Sia II (STX), ST8Sia IV (PST), neural cell adhesion molecule proteins (NCAMs), PSTD, PBR, degree of polymerization (DP), chemical shift perturbation (CSP)

## Abstract

Polysialic acid (polySia) is an unusual glycan that posttranslational modifies neural cell adhesion molecule (NCAM) proteins in mammalian cells. The up-regulated expression of polySia-NCAM is associated with tumor progression in many metastatic human cancers and in neurocognitive processes. Two members of the ST8Sia family of α2,8-polysialyltransferases (polySTs), ST8Sia II (STX) and ST8Sia IV (PST) both catalyze synthesis of polySia when activated cytidine monophosphate(CMP)-Sialic acid (CMP-Sia) is translocate into the lumen of the Golgi apparatus. Two key polybasic domains in the polySTs, the polybasic region (PBR) and the polysialyltransferase domain (PSTD) areessential forpolysialylation of the NCAM proteins. However, the precise molecular details to describe the interactions required for polysialylation remain unknown. In this study, we hypothesize that PSTD interacts with both CMP-Sia and polySia to catalyze polysialylation of the NCAM proteins. To test this hypothesis, we synthesized a 35-amino acid-PSTD peptide derived from the ST8Sia IV gene sequence and used it to study its interaction with CMP-Sia, and polySia. Our results showed for the PSTD-CMP-Sia interaction, the largest chemical-shift perturbations (CSP) were in amino acid residues V251 to A254 in the short H1 helix, located near the N-terminus of PSTD. However, larger CSP values for the PSTD-polySia interaction were observed in amino acid residues R259 to T270 in the long H2 helix. These differences suggest that CMP-Sia preferentially binds to the domain between the short H1 helix and the longer H2 helix. In contrast, polySia was principally bound to the long H2 helix of PSTD. For the PSTD-polySia interaction, a significant decrease in peak intensity was observed in the 20 amino acid residues located between the N-and C-termini of the long H2 helix in PSTD, suggesting a slower motion in these residues when polySia bound to PSTD. Specific features of the interactions between PSTD-CMP-Sia, and PSTD-polySia were further confirmed by comparing their 800 MHz-derived HSQC spectra with that of PSTD-Sia, PSTD-TriSia (DP 3) and PSTD-polySia. Based on the interactions between PSTD-CMP-Sia, PSTD-polySia, PBR-NCAM and PSTD-PBR, these findingsprovide a greater understanding of the molecular mechanisms underlying polySia-NCAM polysialylation, and thus provides a new perspective for translational pharmacological applications and development by targeting the two polysialyltransferases.

## 1. Introduction

The α2-8-linked polysialic acid (polySia) glycans are oncodevelopmental, tumor-associated cell surface antigens that covalently modify the neural cell adhesion molecule (NCAM) proteins in mammalian cells. PolySia-NCAMs play key roles in modulating cell-cell interactions during embryonic neural development, synaptic plasticity, synaptogenesis, neural stem cell proliferation, differentiation and tumor metastasis [1,2,3,4,5]. The same polySia-NCAM proteins are also of critical importance in neurodevelopment, learning memory, and intestinal development in neonatal piglets [6,7].

Synthesis of polySia is catalyzed by two polysialyltransferases (polySTs), designated ST8Sia II (STX) and ST8Sia IV (PST). The finding that polyST gene “knock-down” experiments negates events associated with tumor cell dissemination indicates that polySTs are validated targets for potential drug therapies [8,9,10,11,12,13]. It is also known that cytidine monophosphate (CMP)-sialic acid (CMP-Sia), the activated nucleotide sugar of N-acetylneuraminic acid (Neu5Ac; Sia) [14,15,16,17,18,19], is required by the polySTs for biosynthesis of polySia [9,13,20,21]. Accordingly, it has been proposed that ST8Sia II/IV may interact with CMP-Sia and polySia in the lumen of the Golgi apparatus to catalyze synthesis of the polySia-NCAM protein [20,21,22,23,24,25].

**Figure 1:**
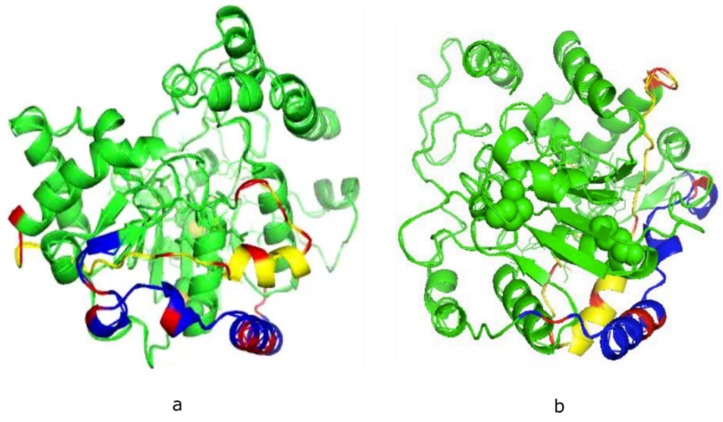
Theoretical models of human ST8Sia II (**a**) and ST8Sia IV (**b**) developed using the Phyre2 server. The basic residues in polysialyltransferase domain (PSTD, yellow) and polybasic region (PBR, blue) are labeled in red. The shortest distance between PBR and PSTD is 4–5 Å.

Neither X-ray crystal or NMR-derived structures of the mammalian polySTs have been reported, due in part to the presence of a large number of hydrophobic amino acids in the membrane environment, and random domains in these enzymes. This has made it difficult to obtain adequate 3-D crystal structures. Encouragingly, the predicted 3-D structures of ST8Sia II (Figure 1a) and ST8Sia IV (Figure 1b) have been confirmed to be accurate 3-D models [26,27]. However, in order to study the dynamics and interactions of biomolecule-biomolecule or biomolecule-ligand, it is necessary to obtain 3-D solution structure of the specific biomolecule or peptide.

Based on mutational analysis of ST8Sia II and ST8Sia IV, two polybasic motifs, designated the polysialyltransferase domain (PSTD) of 32-amino acids [28] and the polybasic region (PBR) *o*f 35-amino acids [29] within both of the polySTs were discovered to be essential for polyST activity, leading to polysialylation of the NCAM protein. Accordingly, two similar hypotheses were proposed to describe the role of PSTD and PBR in catalyzing the specific polysialylation of NCAM. In the first, the interaction within the Golgi of the PSTD peptide domain within ST8Sia II/IV with CMP-Sia, and the polySia moiety of polySia-NCAM glycoprotein, are of key importance, while in the second, it is the interaction between PBR-NCAM, PSTD-CMP-Sia and the PSTD-polySia that are also important [26,27,28,29].

The interaction between PBR and NCAM was confirmed in NMR studies by Colley and colleagues in which the basic amino acid residues in the PBR of ST8Sia IV were found to be bind directly to the acidic patch in NCAM [30]. In our present studies, we initiated structural studies of the interactive binding of PSTD and CMP-Sia, TriSia (DP 3) and polySia using NMR and molecular modeling approaches [31].

Our present studies confirm that CMP-Sia and polySia interact with PSTD with different binding affinities. This finding was further confirmed by comparing their 800 MHz-determined HSQC spectra with PSTD-Sia, PSTD- DP 3 and PSTD-PolySia spectra. Accordingly, we have integrated these new NMR results with previous findings on the interactions of PBR-NCAM, and PBR-PSTD, to describe the interactions of polySia-NCAM polysialylation, which is also based on the interaction of PSTD-CMP-Sia and PSTD-polySia in ST8Sia IV. These molecular interactions also provide a potential perspective for rational drug design to target the polySTs that regulate polysialylation.

## 2. Results and Discussion

### 2.1. Structural Conformational Features of PSTD in ST8Sia IV

The atomic coordinate files for the 32-amino acid PSTD were recently deposited in the Protein Data Bank with the accession code of 6AHZ. The chemical shift assignments were also deposited in the Biological Magnetic Resonance Data Bank, with the accession number 36,207—available online: http://www.bmrb.wisc.edu (accessed on 24 October 2018).

**Figure 2:**
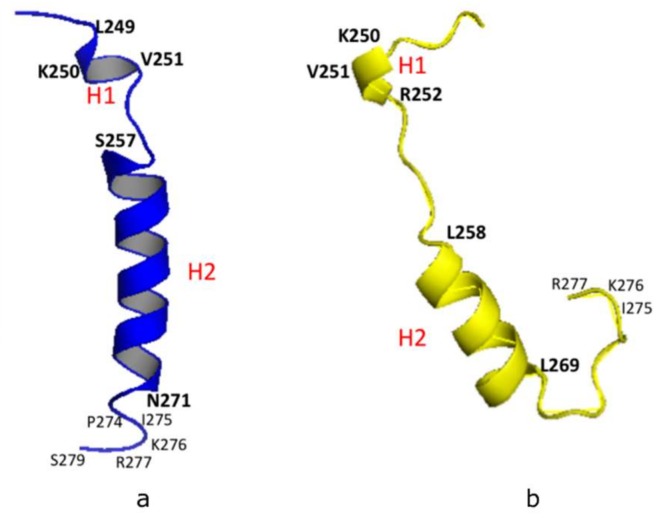
3-D structural backbone models of PSTD in ST8Sia IV, based on NMR spectroscopy (**a**), and the Phyre2 server (**b**). The short helix and the long helix are labeled H1 and H2, respectively.

Our NMR derived 3-D structural model was analyzed using the Pymol software (http://www.pymol.org/) and Discovery Studio Visualizer (Accelrys, Inc., San Diego, CA, USA). The model showed a short helix (**H1**: L249-K250-V251), a longer helix (**H2**: S257-N271), and a short C-terminal loop (C-Loop: I275-K276-R277) (Figure 2a). A similar structural configuration was also found in the predicted PSTD model of ST8Sia IV [26,27] using the Phyre2 sever [31], in which the helical range of the short 3-residue helix (H1) is from K250 to R252. And the 12-residue longer helix (H2) is from L258 to L269 (Figure 2b). In addition, the remaining domains in these two models are all unstructured, except for H1, H2 and the C-loop (Figure 2). These structural comparisons provide further evidence that the NMR-derived PSTD structure is consistent with the predicted PSTD configuration in ST8Sia IV (Figure 2a) [26,27].

**Figure 3:**
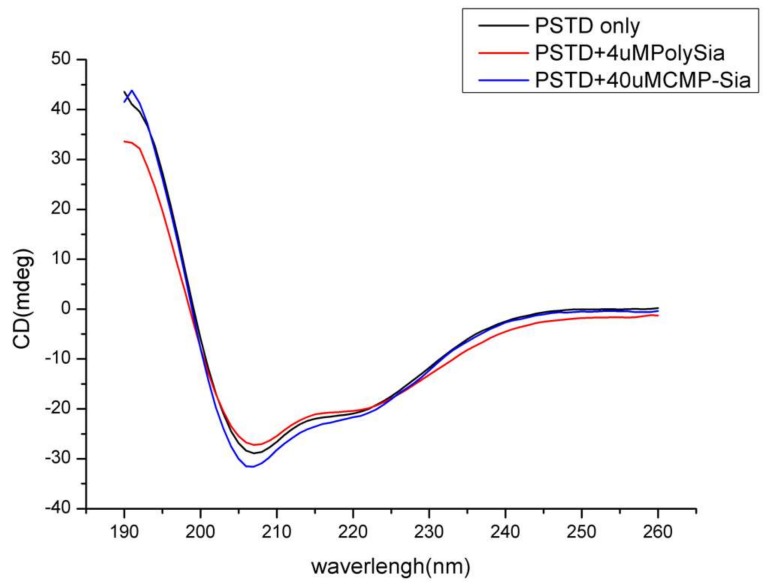
Circular dichroism (CD) spectra of the PSTD peptide from ST8Sia IV in the absence and presence of sialic acid (Sia), cytidine monophosphate-sialic acid (CMP-Sia) and polysialic acid (polySia).

Based on our 3-D derived molecular model (Figure 2a), a total of 18 residues in the 32-amino acid PSTD in ST8Sia IV are α-helices. Therefore, 56% of the residues in the PSTD structure have a helical configuration. As shown in Figure 3, our circular dichroism (CD) spectrum confirms that the main secondary structure of the PSTD peptide remains α-helical, even in the presence of polySia, and less than half of the structure is a random-coil.

### 2.2. Molecular Interaction between CMP-Sia and PSTD in ST8Sia IV

A Summary: Hypothesis and Biosynthesis of PolySia.

The pathway for biosynthesis of CMP-Sia in all eukaryotic cells is initiated in the cytosol with synthesis of Sia, which is translocated to the nucleus where it is activated with CTP to form CMP-Sia [13,21,25,32,33]. CMP-Sia then returns to the cytosol by an unknown mechanism and is subsequently transported into the lumen of the Golgi by a specific CMP-Sia anti-transporter (CMP-SiaTr), where it serves as the activated Sia donor substrate for polysialylation, catalyzed by either ST8Sia II or ST8Sia IV.

Thus, our working hypothesis is an interactive model that describe how the critically essential PSTD peptide moiety within both ST8Sia II and ST8Sia IV interacts with CMP-Sia and the growing polySia chain to catalyze polysialylation of the N-linked glycans on NCAM proteins. This interaction is a processive reaction whereby the activated Sia moiety on CMP-Sia functions as the Sia donor for the Sia to be transferred to the elongating non-reducing terminus of the growing N-linked glycan on the NCAM glycoprotein [22,25,28].

Our hypothesis is thus consistent with, and predicts, a specific molecular interaction between CMP-Sia andST8Sia IV and PSTD-polySia in the Golgi. This supposition has been confirmed in our 800 MHz studies, as described below.

The interaction between protein–protein or a protein–ligand was confirmed in our studies using two-dimensional ^1^H-^15^N HSQC NMR studies, which correlates the ^15^N frequency (chemical shift) with the directly attached ^1^H [34]. For a uniformly ^15^N-labeled protein, the ^1^H-^15^N HSQC spectrum showed chemical shifts of amide ^15^N and ^1^H for every single amino acid from cross peaks [34,35,36,37,38,39,40,41,42,43,44,45,46,47,48,49,50,51,52,53,54,55]. Because chemical shifts of amides in a protein or peptide are very sensitive to environmental changes including pH, temperature or binding of other ligands, the extent of strong or weak interactions of protein-protein or peptide-ligand interactions are reflected by large or small chemical shift perturbations (CSP) [35]. Generally, CSP of a binding complex is determined through the minimum deviation between each position of the free and the complexed peak in the ^1^H-^15^N HSQC spectra [28]. Chemical shift changes can be observed when the CSP value of an amino acid is larger than 0.02 [35].

**Figure 4:**
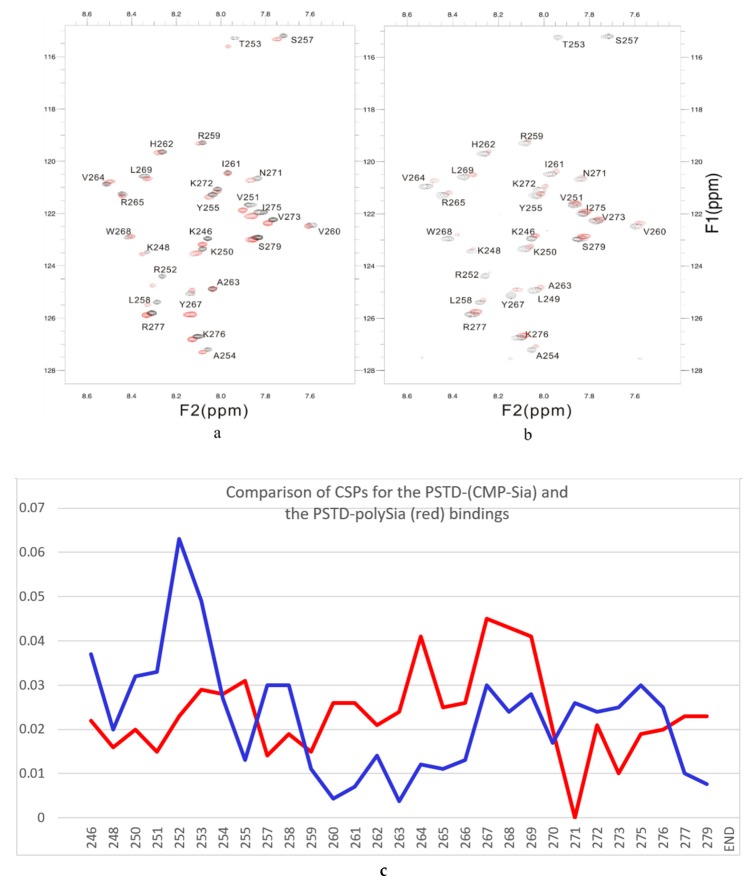
The overlaid ^1^H-^15^N HSQC spectra of PSTD in ST8Sia IV at 800 MHz in the absence (black) and presence (red) of CMP-Sia (**a**) and polySia (**b**). The CSP values for the PSTD-CMP-Sia interaction are shown in blue, and the PSTD-polySia interactions in red (**c**).

Accordingly, to confirm a specific interaction between CMP-Sia and PSTD, the overlaid 2D ^1^H-^15^N HSQC spectra were obtained in the presence and absence of polySia. As showed in Figure 4a, chemical-shift perturbations were evident in the three amino acid segments, K246-A254, S257-L258, and G266-R277, which are primarily located in the N-terminus region of PSTD (K246-A254), the N-terminus of the long helix H2 (S257-L258), and the C-terminus of PSTD (G266-R277).

Among these amino acids, all seven basic residues (K246, K250, R252, H262, K272, K276, R277) and one non-basic residue (I275) are located in the N-terminus, middle and C-terminus of PSTD, as confirmed by site-directed mutagenesis to be critically important for polysialylation of NCAM [28]. However, our NMR findings (Figure 4) further suggest that other non-basic amino acid residues, viz. V251, T253, A254, S257, L258, N271, and V273 are also likely required for polysialylation of the NCAM protein. These NMR results further support our earlier studies showing that the polysialyltransferase activity of ST8Sia IV was not solely dependent on increasing the basicity of PSTD [28].

### 2.3. Specificity of the Interaction of PSTD-Sia, PSTD- DP3, PST- CMP-Sia and PSTD-polySia

While neither free Sia nor DP 3 are normally found in the Golgi, they remain relevant for comparing their functional interaction with PSTD and the polydisperse chains of polySia with DP’s extending up to >400 Sia residues [56]. Accordingly, we compared the specificity of the interaction of PSTD-CMP-Sia with the interaction of PSTD-Sia, PSTD-DP3 and PSTD-polySia.

**Figure A1:**
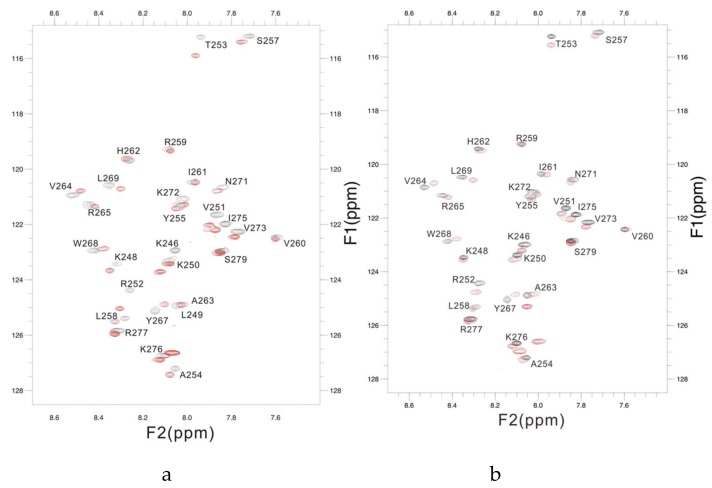
The overlaid ^1^H-^15^N HSQC spectra of PSTD of ST8Sia IV in the absence (black) and presence (red) of 1 mM (**a**) and 1mM DP3 (**b**).

As shown in Table 1, Figure A1a in Appendix A, the largest chemical-shift perturbations (CSP > 0.02) are displayed in three amino acid residual ranges, K246-A254, S257-L258, and G266-R277 for the PSTD-Sia interaction and the two residual ranges from K246 to T253, and G266 to T270 for the PSTD-DP 3 interaction.

**Figure A2:**
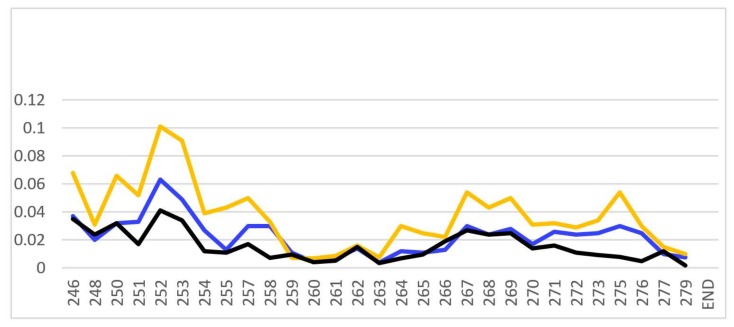
Comparison of the CSPs for binding of PSTD-CMP-Sia (blue), PSTD-Sia (yellow), and PSTD-DP 3 (black) based on the overlaid ^1^H-^15^N HSQC spectra of their interactions.

A common feature is that the larger CSP values (CVP > 0.02) are principally distributed in the N-terminus region of PSTD for the interactions with the three sialyl derivatives. The short helix (L249-V251) is also involved in this amino acid range (Table 1; Figure A1). Although the largest CSP values of the three sialyl interactions are all distributed in the same amino acid residues (V251-A254), the CSP values for the PSTD-Sia interaction are considerably larger than for the interactions between PSTD-CMP-Sia and PSTD-DP 3 (Figure A2). Based on a comparison of the three curves in Figure A2, we conclude that the binding site for the interaction between PSTD-CMP-Sia is between PSTD-Sia and the binding site of PSTD-DP 3.

Also notable from these NMR findings is the interaction between PSTD-Sia, but not PSTD-CMP-Sia, the former showing significant changes in the chemical shift values for amino acid residues V264, Y267, W268, and L269 for PSTD-Sia, but not for PSTD-CMP-Sia. Accordingly, these differences suggest that the configuration of the long H2 helix may be more stable in the interaction between PSTD-CMP-Sia than between PSTD-Sia or between PSTD-DP 3.

### 2.4. Interaction between PSTD and PolySia

The overlay of the 2D 1H-15N HSQC spectra of PSTD in the absence and presence of polySia are shown in Figure 4b. The following key findings are noted:

1. The large chemical-shift perturbations (CSP>0.02) are most prominent in the N-terminus amino acids K248 to A254, for the PSTD-polySia interaction, and are less than for the interactions between PSTD-CMP-Sia, PSTD-Sia and PSTD-DP3 (Figure A2). The CSP values for amino acids V260-T270 are also much greater for the interaction between PSTD-polySia than for the interactions between PSTD-CMP-Sia and PSTD-DP 3 (Table 1; Figure 4c). This finding is consistent with a conformational change in the long H2 helix in PSTD, following its interaction with polySia.
2. These findings also show that the CSP values for the PSTD-polySia interaction in the C-terminus region (from 270 to S279) show small chemical shift perturbations (CSP < 0.02), which are less than for the interaction between PSTD-CMP-Sia and PSTD-Sia (Figure 4c, Figure A2). These results indicate that the predominantly small CSP values are located in the non-structural domains, while the larger CSP values are located on the long H2 helix for the interaction between PSTD-polySia (Figure 4c).
3. The amide cross-peak intensities for the 20 amino acid PSTD peptide were significantly decreased after interaction with polySia. The binding interaction between PSTD and polySia was characterized by broadening NMR signals that appeared in most amino acid residues except V251, V273, I275, K276, and R277 (Figure 4b and Table 1). This suggests a very slow conformational exchange or slow motion during the PSTD-polySia interaction. It is reasonable to expect that a decrease in the rate of tumbling occurs when a significantly larger ligand, such as the polydisperse polySia chains are bound to a protein or peptide [57,58,59,60,61]. The polySia-PSTD interaction is particularly evident, as seen in both the large chemical-shift perturbations, and the significant decrease in cross-peak intensity of amides that are observed in the 4 amino acid residues, V264, Y267, W268 and L269, consistent with the occurrence of both a conformational change and a slower motion in the C-terminus of the long H2 helix.

Because neither Sia nor oligo-polySia exists free in the Golgi [28,29], there is no interaction between either of these two sugars and the polySTs. Therefore, CMP-Sia in the Golgi must interact first with PSTD region within the polySTs to initiate polysialylation on the N-glycan chains covalently linked to NCAM [12,13,22,25]. We determined this is evident by the slowing of the chemical exchange seen for the interaction between PSTD-CMP-Sia, which is slowed as elongation of the polySia chains occurs while remaining covalently linked to the N-glycan chains on the NCAM glycoprotein. This elongation reaction continues until polySia chains with DP’s exceeding 400 Sia chains is completed [56].

The level of CMP-Neu5Ac in the Golgi has been estimated to be ~0.03–0.05 mM [62]. But importantly, this level is dynamic, and will change depending on the extent of sialylated glycans that are being synthesized at any given time. In our studies, we found no difference in the NMR chemical shift and peak intensities using 0.1 and 1 mM CMP-Sia for the interaction studies of PSTD with CMP-Sia, Sia, DP 3.

### 2.5. Functional Importance of the Degree of Polymerization (DP) of α2,8-linked PolySia Chains Covalently Modifying NCAM Proteins Related to Present Studies

The polySia-NCAM proteins are best understood as structurally unique, oncodevelopmental, tumor-associated antigen that plays a key role in modulating cell-cell interactions, principally during embryonic neuronal development, neural plasticity and tumor metastasis [5,13,25,33]. As such, the DP of polySia on N-CAM is a key structural feature hypothesized to be of critical importance in regulating N-CAM associated functions. For example, as an anti-adhesive glycotope, re-expression of the polySia glycan on several human cancers is postulated to attenuate the adhesive property of NCAM, thus facilitating cell detachment and tumor metastasis. It was, therefore, of key importance to be able to accurately determine the DP of polySia on human cancer cells, as described below.

Several significant structural studies have been published providing insight into the importance of the DP of polySia in biochemical, physiological and molecular neurobiological processes. Among others, these studies include the dependency of the DP of polySia binding to neurotrophic factors, their interactions with lactoferrin, their regulation of fibroblast growth factor 2 (FGF) to modulate cell growth, and the cytotoxicity of histones [63,64,65,66,67,68,69,70].

While one study showed the interaction of oligoSia with a protein was as uniquely short as DP 5 for binding to endo-N-acylneuraminidase (endo-NF) [69], others studies have shown that the DP of polySia on human cancer cells and leukocytes are longer, extending to DP’s ~40 and considerably higher [23,56].

DP studies confirming these considerably higher DP values were only made possible following the development of a new experimental strategy to accurately determine the DP of polySia chains that avoided acid catalyzed hydrolysis of the chemically labile, internal α2,8-linked polySia chains that covalently modify the NCAM protein [56].

Unexpectedly, significant new information that emerged from the development of this contemporary experimental method was two-fold. First, expression of the polySia chains expressed on the cell surface of the human *Neuro2a* tumor cells were extremely polydisperse, with sub-populations in the DP range 150–180, and even extending to DP’s ~430 [56].

Second, while polySia is well known to modulated neuronal development [6,13,21,25], the first demonstration that polySia was expressed on primary human natural killer (NK) cells and murine leukocytes, and could modulate immune responses, was shown by Drake et al. [23]. Specifically, these studies revealed two significant new findings: (1) the DP of polySia chains on human NK cells was responsive to their activations state by interleukin 2 (IL-2) and; (2) IL-2 activation resulted in an increase expression of a polydisperse subpopulation of polySia chains (DP 11-140) and an even higher subpopulation of polySia chains with DP’s ranging from 141 to more than 400 [23].

It is, therefore, evident from these two unanticipated findings showing significant polydispersity in the length of polySia on relevant human cancer cells *(Neuro2A*) [56], and human NK cells [23], that the DP of the polySia chains are not restricted to only short chains (DP ~40), which was reported, as noted above, but can be markedly and extensively longer. Furthermore, of significance, is the new finding that the DP of polySia can be modulated, as in the case of NK cells, by their state of activation by IL-2.

Importantly, these new findings thus raise the fundamentally important question: “Is it the polydispersity in the length of the polySia moiety on the NCAM protein that helps explain how polySia can modulate such a surprisingly myriad of cell-cell adhesive and cell migration interactions and functions?” [6,8,13,21,24,25,31,33,67,68].

Accordingly, until this question is resolved, an incomplete understanding of crucial cellular processes in glyco-neurobiobiology, immune regulation and cancer metastasis will remain. These molecular events also include other polySia-NCAM related functions, including axon path finding, synaptogenesis, neural plasticity, cell signaling/cytokine responses, myelination, immune responses, neural stem cell (NSC) proliferations and differentiations, and neurocognitive function including learning and memory.

## 3. Materials and Methods

### 3.1. Preparations of Samples for NMR Studies

To determine the 3-D structure of PSTD using NMR spectroscopy, we synthesized a 35 amino acid peptide (L245-S279 from the gene sequence encoding the human CMP-N-acetylneuraminate-α-2,8-polysialyltransferase, ST8Sia IV, containing the 32-amino acid PSTD (from residue K246 to R277) sequence. The sequence of the 35-amino acid-PSTD peptide (L245-S279) is as follows: 245LKNKLKVRTAYPSLRLIHAVRGYWLTNKVPIKR277PS279.

The 35 amino acid-PSTD from ST8Sia IV was chemically synthesized by DG Peptides (Hangzhou City, Zhejiang Province, China). Its molecular weight was determined to be 4117.95 and its purity established to be 99.36%.

CMP-Sialic acid (CMP-Sia: C_20_H_29_N_4_O_16_P_2_Na), sialic acid (Sia: C_11_H_19_NO_9_), DP 3: C_33_H_50_N_3_O_25_Na_3_), and polySia were purchased from Santa Cruz Biotechnology. The molecular weight of CMP-Sia, Sia and DP3, are 636.43, 309.11, and 957.73, respectively.

The DP of polySia, when accurately determined in the absence of acidic conditions, which occurs in the DMB method for determining the chain length of polySia, is polydisperse, with DP’s ranging from ~ 5 to > 400 Sia residues [56]. The molecular weight of the sodium salt of the polySia/”Colominic acid” (Cat. No. CAS 70431-34-4: sc-239576, purified from *E. coli K1*) used in this study, while polydisperse, is ~31 KDa, with the average DP of ~ 95. In the present study, this sample is designated as “polySia” [56].

For both the 1-D and 2-D NMR experiments, the concentration of the 35 amino acid-PSTD peptide was 2.0 mM. The concentration of CMP-Sia, Sia and DP3 was 1 mM. For all of the NMR studies, the concentration of polySia was (0.1 mM), which was dissolved in 25%TFE (*v/v*), 10% D_2_O (*v/v*), and 65% (*v/v*) 20 mM phosphate buffer (pH 6.7). Following this, 2-Dimethyl-2-silapentane-5-sulfonic acid (DSS) was added to all samples to serve as a reference standard.

### 3.2. Circular Dichroism (CD) Spectroscopic Studies

All CD spectra were recorded in the far-UV region (190–260 nm) at 25 °C in a 0.02 cm path-length quartz cell on a Chirascan spectropolarimeter, Beverly, MA, USA.

The concentrations of CMP-Sia, polySia and the 35 amino acid-PSTD were 40 µM, 4 µM and 80 µM, respectively and were dissolved in 20 mM 25%TFE/75% 20 mM phosphate buffer (pH 6.7), respectively.

### 3.3. NMR Spectroscopy

All NMR spectra were recorded at 298 K using an Agilent DD2 800 MHz spectrometer equipped with a cold-probe in the NMR laboratory at the Guangxi Academy of Sciences. Water resonance was suppressed using pre-saturation. NOESY mixing times were set at 300 msec while the TOCSY experiments were recorded with mixing times of 80 msec. All chemical shifts were referenced to the internal DSS signal set at 0.00 ppm for proton and indirectly for carbon and nitrogen [71,72]. Data were typically apodized with a shifted sine bell window function and zero-filled to double the date points in F1 prior to Fourier transformed. NMRPipe [72] and CcpNmr (www.ccpn.ac.uk/v2-software/analysis) were used for data processing and spectral analysis, respectively. Spin system identification and sequential assignment of individual resonances were carried out using a combination of TOCSY and NOESY spectra, as previously described [72,73], and coupled with an analysis of 1H-15N and ^1^H-^13^C HSQC for overlapping resonances. In order to identify specificity of the PSTD-ligand binding, the chemical shift perturbation (CSP) of each amino acid in the PSTD was calculated using the formula:

CSP = [(Δδ^2^_NH_ + (Δδ_N_/5)^2^)/2]^1/2^ (1)

where Δδ_N_ and Δδ_NH_ represent the changes in ^15^N and ^1^H chemical shifts, respectively, upon ligand binding [34,55].

### 3.4. Structural Calculation and Analysis of the PSTD from ST8Sia IV

Structural calculations and NOE assignments were carried out simultaneously using the CNS program [55,72] and ARIA2 [47]. Backbone dihedral angle restraints (Φ and Ψ angles) were derived using the DANGLE program incorporated in the CcpNmr suite [72]. A total of 100 structures were calculated and the 20 structures with the lowest total energy were selected to carry out a refinement procedure in water. The atomic coordinates of the peptides have been deposited in the Protein Data Bank with the accession code of 6AHZ, and the chemical shift assignments were deposited in the Biological Magnetic Resonance Data Bank with the accession number of 36,207 (http://www.bmrb.wisc.edu). The protein structure ensemble was displayed and analyzed with the Pymol software (http://www.pymol.org/) and Discovery Studio Visualizer (Accelrys, Inc., San Diego, CA, USA).

### 3.5. Structural Predictions of ST8Sia II and ST8Sia IV

For the structural calculations of the wild type and mutants of ST8Sia II and ST8Sia IV, the crystal structure of ST8Sia III was selected as the template (code: c5bo6B) based on Server Phyre2 server [31]. The predicted structures are based on heuristics to maximize confidence (100%), and the sequence identity was 26%. All structural models were displayed by PyMol software (https://pymol.org/2/).

## 4. Conclusions

Volkers et al. [61] proposed that PSTD and PBR together form an extended basic groove for substrate recognition for the processive synthesis of the growing polySia chain. Bhide et al. [30] further suggested that a possible PBR-PSTD interaction might relate to the basic surface of the polySTs for substrate recognition and/or polySia chain elongation. In our recent molecular modeling study, we found that the PBR and PSTD domains are remarkably close, the shortest distance between them being 4–6 Å (Figure 1) [27]. This finding is in accord with the previous report of Colley and colleagues [30]. Therefore, it is a reasonable supposition that an intramolecular interaction exists between PBR and PSTD in ST8Sia IV, based on the short distance between PBR and PSTD. This intramolecular interaction may thus stabilize the conformation of ST8Sia IV, and modulate polysialylation and autopolysialylation, catalyzed by ST8Sia II and ST8Sia IV [74,75].

In an impressive series of studies, Colley and colleagues among others, have provided evidence that neuropilin-2 (NRP2) and E-Selectin Ligand-1 (ESL-1) were identified as targets for polysialylation in murine microglia and human THP-1 macrophages, showing a striking convergence in the regulation of these two polysialylated acceptors during an inflammatory activation [76,77]. In addition, polySia-SynCAM 1 was identified in a subpopulation of NG2 cells, and the presence of polySia-NRP2 was established in microglia. Further, the dynamic rearrangement of polySia from retention in the Golgi compartment towards recruitment to the cell surface in response to relevant stimuli in the onset of myelination, and in the regulation of microglia activation, were also determined [76]. These findings clearly establish there are multiple targets for polysialylation catalyzed by ST8Sia II and ST8Sia IV other than NCAM proteins alone. The molecular interactions between the polySTs and these additional targets will require further studies to understand the molecular significance and biological consequences of their polysialylation.

In summary, results from our current NMR studies, and previous findings on the DP of polySia chains and the cooperative interactions between PSTD-CMP-Sia, PSTD-polySia, PBR-NCAM, and PBR-PSTD [26,27,28,29,30,63,78] now provide greater insight into key molecular processes regulating the importance of these interactions in modulating NCAM polysialylation, which underlie many of the neuronal and immune system development and cancer metastatic events in human health and disease.

## Figures and Tables

**Table 1:** Major features of the overlaid ^1^H-^15^N HSQC spectra of PSTD in the absence and presence of Sia, TriSia, CMP-Sia and polySia.

Ligand Interacting with PSTD	Amino Acid Residues Displaying Large Chemical Shift Perturbation (CSP > 0.02)	Amino Acid Residues Displaying the Largest CSP Values	Amino Acid Residues Exhibiting Significant Decrease in Peak Intensities
polySia	R252-P256 V260-T270	A263-T270	20 residues: K246, K248, K250, R252, T253, A254, Y255, S257, L258, R259, I261, H262, A263, V264, R265, Y267, W268, L269, N271, K272.
CMP-Sia	K246-A254, P256-L258, Y267-L269, N271-R277	V251-A254	None
Sia	K246-L258, V264-R277	V251-A254	None
DP 3	K246-T253, G266-T270	V251-A254	None

## Abbreviations

polySia	α2,8-linked polysialic acid
Sia	sialic acid (Neu5Ac; N-acetylneuraminic acid)
DP3	TriSia
CMP-Sia	cytidine monophosphate-sialic acid
NCAM	neural cell adhesion molecule proteins
polySTs	polysialyltransferases (ST8Sia II (STX) & ST8Sia IV (PST)
PSTD	polysialyltransferase domain
PBR	polybasic region
DP	degree of polymerization
E. coli	Escherichia coli
